# Beyond the Central Nervous System: Neisseria meningitidis as an Unusual Cause of Urogenital Infection

**DOI:** 10.7759/cureus.102551

**Published:** 2026-01-29

**Authors:** Faisal Alhetheel, Bandar A Alazmi, Bandar S Alshammari, Abdulkarim F Alhetheel

**Affiliations:** 1 College of Pharmacy, King Saud University Medical City, Riyadh, SAU; 2 Department of Pathology, King Saud University Medical City, Riyadh, SAU

**Keywords:** chronic prostatitis, maldi-tof ms, neisseria meningitidis, saudi arabia, urogenital infection

## Abstract

*Neisseria meningitidis* is a well-known cause of invasive diseases such as meningitis and septicemia, but is increasingly recognized as an emerging urogenital pathogen. We report the case of a 36-year-old man who presented to a tertiary hospital in Riyadh, Saudi Arabia, with persistent dysuria and purulent urethral discharge. Despite initial treatment for nonspecific urethritis and subsequent management for chronic prostatitis with levofloxacin and azithromycin, his symptoms persisted. Although standard multiplex polymerase chain reaction (PCR) assays for common sexually transmitted infections were negative, specialized culture on Thayer-Martin medium followed by VITEK (bioMérieux, France) mass spectrometry identified* N. meningitidis*. This case highlights the diagnostic challenges associated with meningococcal urogenital infections, which can mimic gonorrhea yet evade detection by routine molecular sexually transmitted infection panels.

## Introduction

The genus *Neisseria* contains two primary human pathogens: *Neisseria gonorrhoeae* and *Neisseria meningitidis* [1]. While *N. gonorrhoeae* is the classic agent of the sexually transmitted infection gonorrhea, *N. meningitidis* is typically a commensal of the human nasopharynx that occasionally causes life-threatening invasive disease [2]. However, the traditional clinical boundaries between these species are blurring due to the emergence of urogenital-adapted meningococcal lineages [3].

Historically, meningococcal urogenital infections were considered rare, though cases of urethritis have been documented as far back as 1942 [4]. Recently, the emergence of specific clades, such as the ST-11 clonal complex (NmUC), has led to outbreaks of urethritis primarily among heterosexual males [5]. These strains have undergone significant evolutionary adaptation, including the loss of the polysaccharide capsule and the acquisition of the gonococcal aniA gene, which allows for anaerobic growth in the male urethra [1]. 

In Saudi Arabia, the epidemiology of *N. meningitidis* is closely linked to mass gathering events like the Hajj and Umrah pilgrimages [6]. These events facilitate high rates of nasopharyngeal carriage and the potential for orogenital transmission through close contact [6]. This case report describes a patient in Riyadh whose persistent prostatitis was caused by *N. meningitidis*, highlighting the need for vigilance when standard molecular diagnostic arrays fail to identify a pathogen [7].

The emergence of adapted *N. meningitidis* urethritis clade (NmUC) is a potential clinical consideration in the differential diagnosis of urethritis [8]. While *N. gonorrhoeae* is the expected pathogen in purulent urethritis, clinicians must now recognize the potential for "niche-switching," where *N. meningitidis* transitions from a nasopharyngeal commensal to a primary urogenital pathogen. This adaptation is driven by the loss of the polysaccharide capsule, a structure typically required for invasive disease (meningitis) but one that hinders adherence to urethral epithelial cells. Clinically, meningococcal urethritis is indistinguishable from gonococcal infection, often presenting with identical purulent discharge and dysuria; thus, it represents a relevant diagnostic consideration for urologists and general clinicians [9]. However, the primary pitfall lies in modern diagnostics: most standard nucleic acid amplification tests (NAATs) are engineered with high specificity for *N. gonorrhoeae* DNA and will return a negative result for *N. meningitidis*. This "diagnostic escape" can lead to inappropriate labeling of the infection as "non-specific urethritis," resulting in treatment failure, persistent symptoms, and potential progression to chronic prostatitis.

## Case presentation

Initial presentation (day 1)

A 36-year-old male presented to the urology department at a tertiary care hospital in Riyadh, Saudi Arabia, with a one-month history of dysuria and purulent urethral discharge. Physical examination confirmed significant purulent discharge from the urethra. A presumptive diagnosis of nonspecific urethritis was made. The initial treatment plan consisted of oral azithromycin (500 mg daily for 3 days) as empiric therapy for nongonococcal urethritis. Laboratory investigations included urine and urethral discharge culture and sensitivity (C&S), a complete urine examination (CUE), and a sexually transmitted infection (STI) multiplex array. The CUE results were normal except for elevated red blood cells (Table 1). Crucially, the STI multiplex array did not detect common pathogens. The sample was tested for sexually transmitted infections, including the following organisms: Herpes simplex virus I, Herpes simplex virus II, *Chlamydia trachomatis, Haemophilus ducreyi, Mycoplasma genitalium, Mycoplasma hominis, Neisseria gonorrhoeae, Treponema pallidum, Ureaplasma urealyticum, and Trichomonas vaginalis* [10].

**Table 1:** Clinical laboratory parameters and findings The table summarizes the diagnostic progression. CUE included dipstick testing (for leukocyte esterase) and microscopic analysis (for red blood cells). The negative molecular results, STI PCR, despite positive cultures on selective media, illustrate the diagnostic gap. CUE: complete urine examination; STI: sexually transmitted infection; PCR: polymerase chain reaction; MALDI-TOF MS: matrix-assisted laser desorption/ionization time-of-flight mass spectrometry

Parameter	Day 1 result	Day 30 result	Reference range	Interpretation
Red blood cells (CUE)	5-10 hpf	3 hpf	0-2/hpf	Hematuria
Leukocyte esterase	Negative	Positive	Negative	Pyuria
STI multiplex PCR	Negative	Not done	Negative	No common STIs
MALDI-TOF MS	Not done	>2.0	<1.7	99.9% confidence *Neisseria meningitidis*

Follow-up (day 3)

Upon completion of the initial azithromycin course, the patient reported no improvement. The diagnosis was revised to suspected chronic prostatitis. The medication regimen was changed to oral levofloxacin 500 mg once daily and azithromycin 500 mg once weekly.

Persistent symptoms (day 30)

After completing the initial treatment course, the patient returned with intensified symptoms, including dysuria, frequent micturition, urgency, and pain localized to the penile, perineal, and suprapubic areas. Examination revealed a tender prostate gland and suprapubic tenderness; therefore, the persistence and progression of symptoms supported a diagnosis of chronic prostatitis.

**Figure 1:**
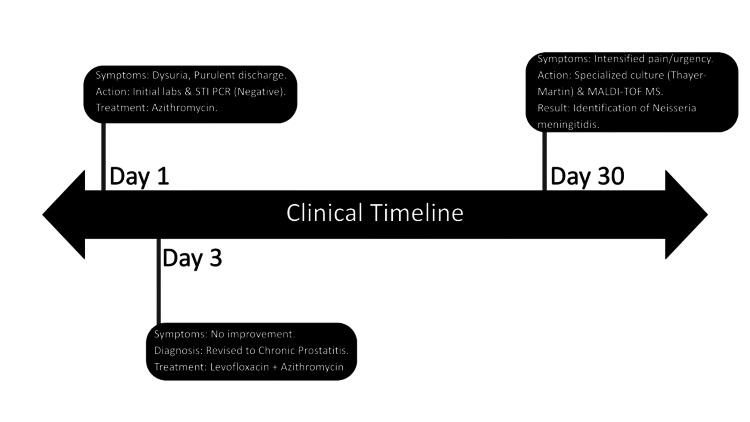
Clinical timeline for the patient STI: sexually transmitted infection; PCR: polymerase chain reaction Image credits: Authors

A comprehensive laboratory panel was requested, including repeat C&S testing of urine and semen, random blood glucose, hepatic and kidney profiles, and vitamin D levels. The repeat CUE demonstrated hematuria and the presence of leukocyte esterase (Table 1). As shown in Figure 1, the clinical progression for this patient is summarized. A continued four to six week course of levofloxacin plus azithromycin following the Day 30 visit was intended to align with chronic prostatitis guidelines once the specific pathogen (*N. meningitidis*) was finally identified via specialized culture and matrix-assisted laser desorption/ionization time-of-flight mass spectrometry (MALDI-TOF MS).

Methods

This case report includes limited demographic and clinical information, such as the patient’s age and relevant clinical timelines, solely for scientific and educational purposes. No direct identifiers were included, such as the patient’s name, initials, medical record number, exact dates of admission or discharge, images, or any other information that could reasonably lead to patient identification. The risk of patient identification was considered minimal, and appropriate measures were taken to preserve patient confidentiality. Verbal informed consent for publication was obtained from the patient, in accordance with institutional practice. Written consent was not required due to the absence of direct identifiers and the minimal risk of identification.

The diagnostic identification was performed at the clinical microbiology laboratory of the Center Hospital in Riyadh. A sample of the purulent urethral discharge was initially tested using a commercial STI multiplex array polymerase chain reaction(PCR) (Vivalytic STI Test, BOSCH), which returned negative results for all standard targets, including *N. gonorrhoeae*.

For phenotypic identification, the urethral swab was introduced into cooked meat medium (CMM) to enrich for bacterial growth. The enriched sample was subsequently subcultured onto the following media (Table 2).

**Table 2:** Type of media used Chocolate agar: As an enriched medium, it supports the growth of fastidious organisms that require specific growth factors found in lysed blood. Thayer-Martin agar: This is a selective medium specifically designed to isolate *Neisseria* species by inhibiting the growth of fungi and other bacteria. Blood agar: A general-purpose enriched medium used to grow a wide variety of organisms and detect hemolytic activity (the breakdown of red blood cells). The fastidious nature of the isolate lowers its growth on unlysed blood. Sabouraud dextrose agar: It is a specialized medium designed primarily for the isolation and cultivation of yeasts and molds. MacConkey agar: This confirms that the organism is not a standard enteric Gram-negative rod, further narrowing the identification to fastidious species like *Neisseria meningitidis or Neisseria gonorrhoeae*.

Type of media	Note	Result
Chocolate agar	Enriched media support the growth of fastidious organisms	Moderate growth
Thayer-Martin agar	Selective media for *Neisseria* species	Moderate growth
Blood agar	General enriched media for most organisms	Mild growth
Sabouraud dextrose agar	Selective media for fungi	No growth
MacConkey agar	Selective media for Gram-negative organisms	No growth

Growth of grayish-white colonies was observed on Chocolate and Thayer-Martin agar media. A smear from the colonies grown on the cultured media was prepared, and a Gram stain was performed. The Gram stain showed Gram-negative diplococci. The oxidase test was also performed and showed oxidase positivity. The isolate was sampled and identified using MALDI-TOF MS via the VITEK MS (bioMérieux, France) system. The VITEK MS utilizes an advanced spectra classifier algorithm to analyze species-specific protein profiles, considering approximately 1300 data points to achieve high resolution. The system confirmed the isolate as *N. meningitidis* with an increased result (Table 1).

The hallmarks of *N. meningitidis* can be easily identified via the carbohydrate utilization test and NAATs, and it is distinguished by a capsule that *N. gonorrhoeae* lacks, as it uses pili and Opa proteins to adhere to the urethral epithelium (Table 3).

**Table 3:** The biochemistry panel of Neisseria meningitidis The biochemical differentiation of urogenital Neisseria isolates is described in this table. This panel outlines the specific metabolic and enzymatic reactions required to confirm *Neisseria meningitidis*. The identification relies heavily on the CTA sugar tests or rapid carbohydrate utilization tests. While *Neisseria​​​ gonorrhoeae *and *Neisseria meningitidis* both utilize glucose, the ability of this isolate to utilize maltose is the primary biochemical differentiator. Furthermore, the negative results for lactose and sucrose exclude common commensal species that might inhabit the urogenital tract or nasopharynx. This phenotypic panel provides the necessary confirmation when molecular NAAT/PCR assays result in “diagnostic escape” due to high specificity for *Neisseria gonorrhoeae*. CTA: cystine trypticase agar; PCR: polymerase chain reaction; NAAT: nucleic acid amplification test

Biochemical assay	Expected result for *Neisseria meningitidis*	Clinical interpretation
Gram stain	Gram-negative diplococci	Confirms *Neisseria* genus morphology
Oxidase test	Positive	Confirms the isolate belongs to the *Neisseria* genus
Catalase test	Positive	Standard for most *Neisseria* species
Glucose utilization	Positive	Indicates oxidative metabolism of glucose
Maltose utilization	Positive	The definitive hallmark; distinguishes *Neisseria meningitidis* from *Neisseria gonorrhoeae*
Lactose utilization	Negative	Excludes *Neisseria lactamica*
Sucrose utilization	Negative	Excludes *Neisseria sicca*
Gamma-glutamyl aminopeptidase	Positive	Enzymatic marker specific to *Neisseria meningitidis*

## Discussion

This case appears to be an unusual occurrence within the clinical landscape of Saudi Arabia. The isolation of *N. meningitidis* in a patient presenting with symptomatic prostatitis suggests a potential diagnostic gap that warrants further consideration. While most modern laboratories prioritize NAATs for STI screening due to their superior speed compared to traditional culture methods, this case illustrates a scenario where such reliance may be insufficient [11]. However, these assays are designed with high specificity for *N. gonorrhoeae* to avoid cross-reactivity with commensal Neisseria species [12]. Consequently, urogenital-adapted *N. meningitidis* strains, which lack the specific DNA targets used in these arrays, frequently result in false-negative findings [13].

The pathogenesis of this infection likely involves niche switching. *N. meningitidis* urethritis clades (NmUC) often lose their capsule to better adhere to urethral epithelial cells [10]. The acquisition of gonococcal alleles through horizontal gene transfer further enhances their fitness in the genitourinary environment. Orogenital contact remains the most probable route of transmission from the nasopharynx to the urogenital tract [9].

In the context of Saudi Arabia, the prevalence of *N. meningitidis* is influenced by the Hajj and Umrah pilgrimages [7]. While mandatory vaccination has reduced invasive disease, it does not fully eliminate nasopharyngeal carriage, which increases significantly following these mass gatherings [14]. Recent reports from 2024 to 2025 have identified clusters of ciprofloxacin-resistant meningococcal strains associated with travel to the region [15]. The failure of the initial levofloxacin course in this case may be indicative of emerging fluoroquinolone resistance in meningococcal clades, as seen in recent outbreaks.

The initial use of azithromycin was likely based on a presumptive diagnosis of nonspecific urethritis, a common clinical approach when a patient presents with purulent urethral discharge and dysuria. Azithromycin is a macrolide frequently employed as a first-line agent for urethritis because of its broad coverage against common pathogens like *Chlamydia trachomatis *[16]. In this specific case, it was administered while awaiting the results of the initial laboratory investigations and the STI multiplex array.

The shift to azithromycin plus levofloxacin occurred on Day 3 after the patient reported no clinical improvement following the completion of the initial azithromycin course. This lack of response prompted a revision of the diagnosis to chronic prostatitis. Fluoroquinolones like levofloxacin are often selected for suspected bacterial prostatitis because they possess superior pharmacokinetic properties, specifically the ability to achieve high concentrations in prostatic tissue, which is essential for treating deep-seated infections in the gland [17].

The decision to utilize dual therapy, combining levofloxacin and azithromycin, was implemented during the second phase of treatment to provide broader antimicrobial coverage for the revised diagnosis of chronic prostatitis [16].

The patient's treatment included a prolonged course of azithromycin (500 mg daily for the first three days, followed by 500 mg weekly for the remaining 6 weeks), plus a daily dose of 500 mg levofloxacin. Standard protocols for chronic bacterial prostatitis typically recommend 4-6 weeks of therapy rather than extended multi-year courses [17]. 

Study limitations

This report is primarily limited by its retrospective design and the reliance on existing medical records. A key gap is the lack of a detailed sexual history, which prevents a definitive conclusion on the transmission route, such as orogenital contact, from the nasopharynx to the urogenital tract. Additionally, the absence of vaccination records makes it difficult to assess how the patient’s immunization status relates to potential nasopharyngeal carriage or the infection's emergence despite regional vaccination efforts.

Furthermore, the lack of recorded antimicrobial susceptibility testing (AST) means that suspected resistance, such as to fluoroquinolones, could not be laboratory-confirmed. While MALDI-TOF MS provided species-level identification, the study did not include further characterization such as serogrouping, capsule typing, or genomic confirmation. Consequently, these findings are specific to this individual case and have limited generalizability.

## Conclusions

This case suggests that *N. meningitidis* may act as an infrequent urogenital pathogen capable of mimicking the clinical presentation of chronic bacterial prostatitis. A notable observation is the potential for "diagnostic escape," where standard molecular STI panels may not detect meningococcal strains that have adapted to the urethral environment. While this report highlights the utility of combining phenotypic culture on selective Thayer-Martin media with MALDI-TOF MS for identification, these findings are intended to suggest directions for future research rather than to serve as a basis for altering current clinical protocols. Further research is needed to determine the broader clinical implications of these diagnostic challenges.
